# Supplementary material for: Type IV Collagen Is Essential for Proper Function of Integrin-Mediated Adhesion in Drosophila Muscle Fibers
Source: Int J Mol Sci. 2019 Oct 16;20(20):5124. doi: 10.3390/ijms20205124 (PMC6829409; doi:10.3390/ijms20205124)
Supplement: Supplementary file 1 [file ijms-20-05124-s001.pdf]

Supplementary Materials

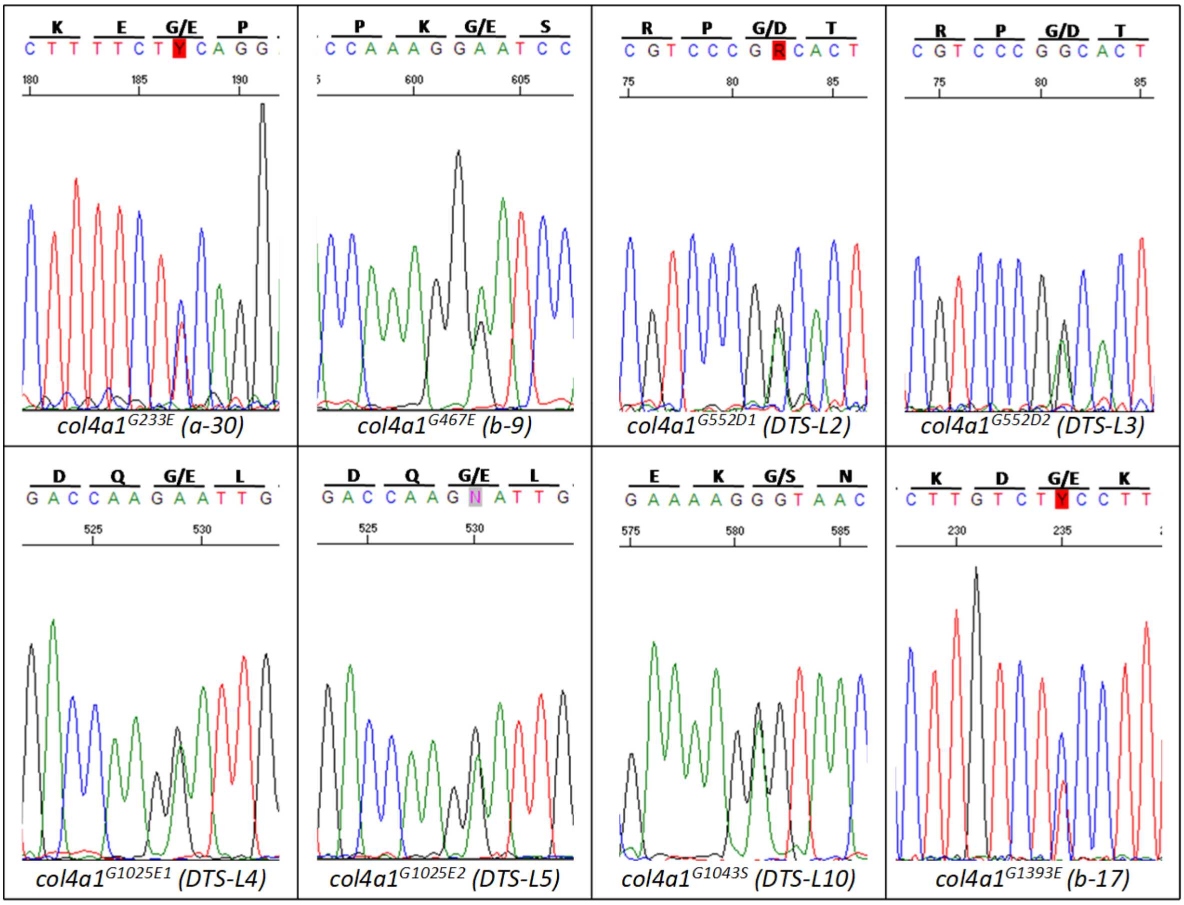

Supplementary Figure S1. Nucleotide sequence of the heterozygous lesions in *col4a1* alleles. Note the isoallelic mutations G552D and G1025E. For a better resolution of the heterozygous site the sequence of the complementary strand is displayed in the *b-17* mutant.

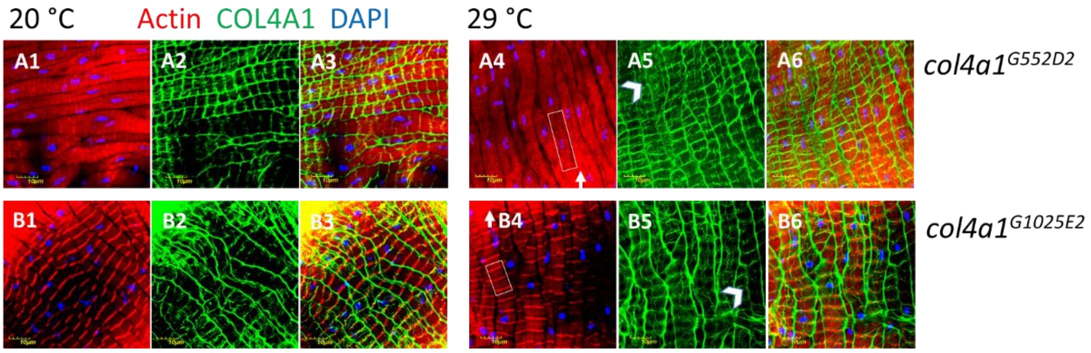

Supplementary Figure S2. Loss of sarcomeres (A4, B4) actin bundle development (white rectangles, A4, B4), actin aggregates (white arrows, A4, B4), irregular COL4A1 deposition (white arrowheads, A5, B5) in the isoallelic lines *col4a1*^{G552D2} and *col4a1*^{G1025E2}. A3, B3, A6, B6: Overlays. Bars, lower left, 10 micrometers.

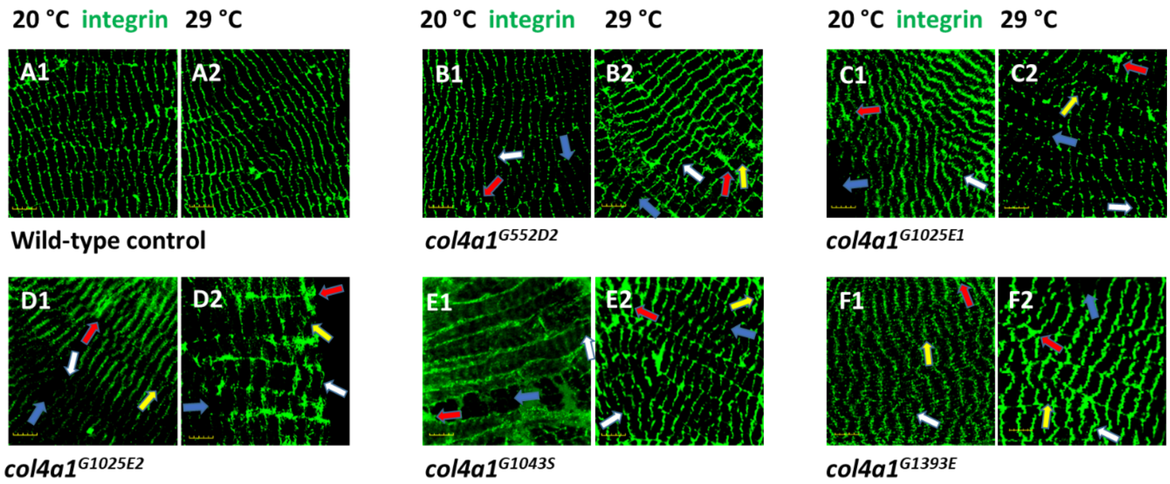

Supplementary Figure S3. Integrin expression in wild-type control (A) and in *col4a1* mutant animals (B-F) at permissive and restrictive temperatures. Streaming of the Z-discs (white arrows), integrin expression within the sarcomeres (yellow arrows), excess integrin expression (red arrows) or deficient integrin deposition (blue arrows) are noted in the mutants (panels B-D). Bars, lower left, 10 micometers.

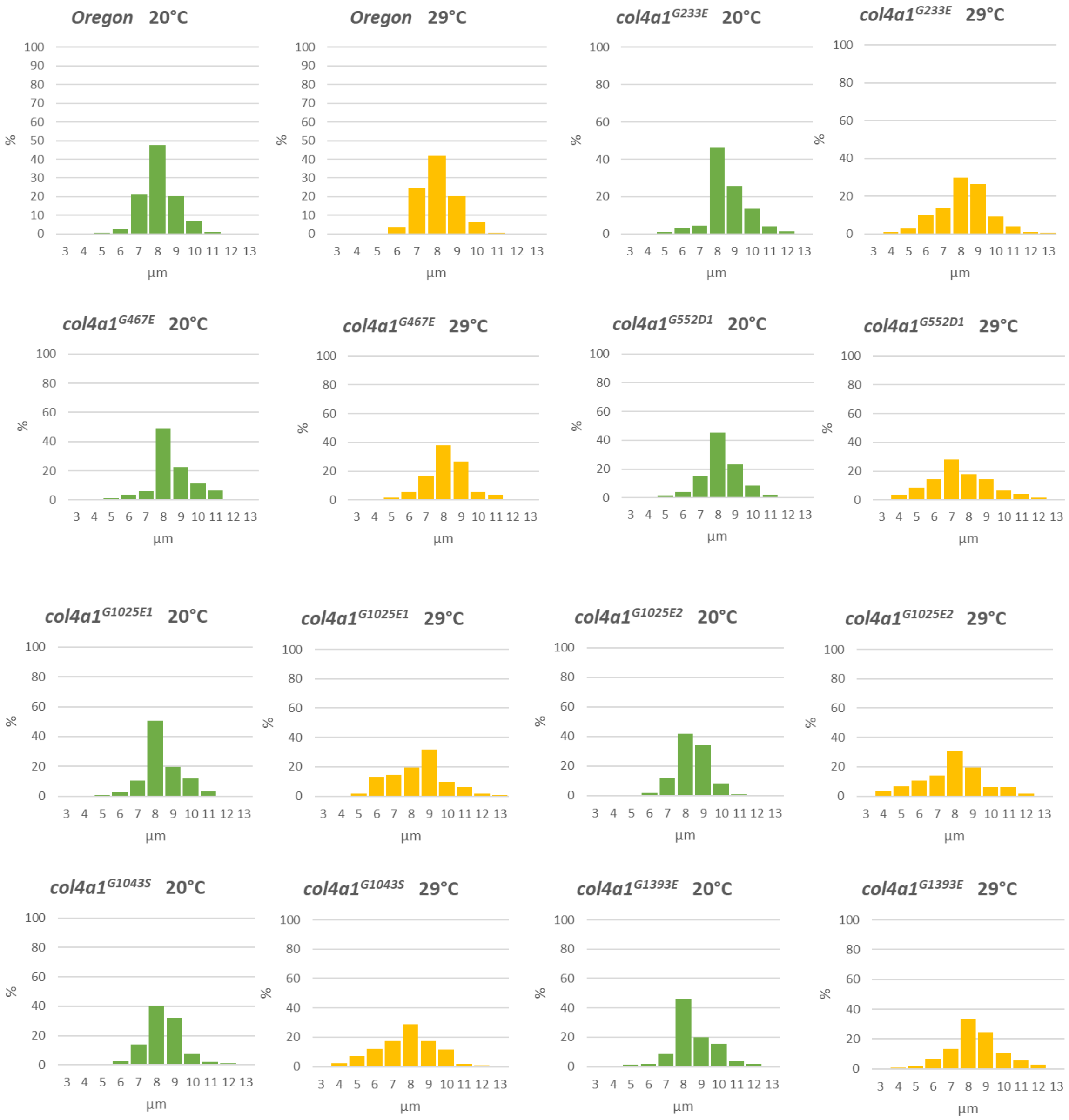

Supplementary Figure S4. Size distribution of muscle fibers in *col4a1* mutants. Diameters of muscle fibers are shifted toward reduced values (yellow columns) in mutants at 29 °C.
